# Association between the accessory gene regulator (*agr*) locus and the presence of superantigen genes in clinical isolates of methicillin-resistant *Staphylococcus aureus*

**DOI:** 10.1186/s13104-019-4166-7

**Published:** 2019-03-12

**Authors:** Hamed Tahmasebi, Sanaz Dehbashi, Mohammad Reza Arabestani

**Affiliations:** 1 Microbiology Department, School of Medicine, Zahedan University of Medical Sciences, Zahedan, Iran; 2 Microbiology Department, Faculty of Medicine, Hamadan University of Medical Sciences, Pajoohesh Junction, Hamadan, Iran; 3 Department of Microbiology, School of Medicine, University of Hamadan, Hamadan, Iran; 4 Brucellosis Research Center, Hamadan University of Medical Sciences, Hamadan, Iran

**Keywords:** Methicillin-resistant *Staphylococcus aureus*, Superantigens, Virulence factors, *agr* locus

## Abstract

**Objective:**

Methicillin-resistant *Staphylococcus aureus* cause to a variety of hard to cure infections. MRSA isolates also, produce an arsenal of virulence factors contribute to severe infections. The aim of this study was to find out the relationship between *agr* locus and presence of *S. aureus* superantigens (SAgs).

**Results:**

Clinical isolates in two groups from two different states of Iran were collected. Antibiotic resistance patterns, *agr* typing, and virulence factor genes prevalence were identified and relationship between them was analyzed using SPSS software version16. Most of the samples were collected from wound 39 isolates in Group 1 and 61 isolates in Group 2. Frequency of MRSA strains was 38.1% in Group 1 and 52.1% in Group 2. Also, the most common resistance among both groups was to penicillin. *agr* positive isolates were detected in 132 isolates of Group 1 and 104 isolates of Group 2. In Conclusion, a significant relationship between the SAgs frequency and *agr* locus in both groups has been indicated. The production of superantigens in *S. aureus* plays an important role in the classification of *agr* locus, and this locus can affect differently in methicillin-resistant strains.

**Electronic supplementary material:**

The online version of this article (10.1186/s13104-019-4166-7) contains supplementary material, which is available to authorized users.

## Introduction

Inappropriate use of antibiotics to treat *S. aureus* infections have led to the development of antibiotic resistant strains. The first cases of methicillin-resistant *S. aureus* (MRSA) were identified in the 1960s, shortly after its introduction into clinical practice [1, 2]. Methicillin resistance is conferred by the *mecA* gene, which encodes a novel penicillin binding protein (PBP2A) [3, 4]. This protein has a reduced affinity for β-lactam antibiotics. The *mecA* gene is carried on a mobile genetic element known as the Staphylococcal Cassette Chromosome *mec* (SCCmec), which can be horizontally transferred between Staphylococcal strains [5].

*Staphylococcus aureus* encodes toxin and superantigens like hemolysins, enterotoxins, exotoxins, exfoloative toxins, toxic shock syndrome toxin-1 (TSST-1) and leukotoxins such as the Panton-Valentine leukocidin (PVL). Different *S. aureus* strains encode different toxins. Exfoliative toxins, TSST-1 and PVL are presented only in some clones [6]. Reduced toxicity can hide the bacteria from the immune system, therefore, facilitate more stable and successful colonization in the host [7]. However, there are a number of undescribed genes in the MRSA strains, which encode virulence factors associated with infections in animals and human. Global regulators such as the accessory gene regulator (*agr*) system, Staphylococcal accessory regulator (Sar) and *S. aureus* exoprotein expression (Sae), have been well characterized which could help bacteria to adapt to a hostile environment [8, 9].

The production of *S. aureus* virulence factors is directly related to methicillin resistance. The *mecA* gene indirectly activates Autoinducer peptides (AIPs), which play an important role in the production of some regulatory factors, biofilms and quorum-sensing (QS) [9]. Beceiro et al. state that methicillin resistance induces cell wall alterations that affect the *agr* quorum-sensing system of the bacteria and consequently reduced virulence in a murine model of sepsis [9, 10].

In this research, MRSA and non-MRSA strains were examined with the aim of investigating the relationship between *agr* regulatory system and virulence factors.

## Main text

### Methods

#### Isolation and identification of *S. aureus*

This cross-sectional study was designed to measure the prevalence of methicillin-resistant *Staphylococcus aureus* among patients and healthcare workers (three hospitals, four clinical laboratories, and two healthcare centers) in *Hamadan* (Group 1) and *Sistan and Baluchistan* (Group 2) during July 2015 and August 2016. A multistage sampling method was used to select areas with different climate. Based on the distribution patterns of antibiotic resistance and different characteristics of the 28 states, the two states with the most differences in climate were selected. Sampling was done by considering the temperature variation index in different seasons and analyzing this index. Clinical specimens were inoculated on sheep blood agar (Merk, Darmstadt, Germany) and mannitol salt agar (Merk, Darmstadt, Germany), and incubated at 35–37 °C for 18 to 24 h aerobically. Biochemical tests were implicated to confirm the suspected isolates [11].

#### Detection of MRSA and determination of antimicrobial susceptibility profile of each isolate

Antimicrobial susceptibility testing was carried out by the Kirby Bauer disc diffusion method according to the Clinical Laboratory Standards Institute (CLSI) guidelines 2017 on Muller Hinton agar (Merk, Darmstadt, Germany). The following drugs were used to determine the antibiotic susceptibility: penicillin (10 U), tetracycline (30 μg), clindamycin (30 μg), gentamicin (30 μg), ciprofloxacin (5 μg), erythromycin (15 μg), chloramphenicol (30 μg), rifampin (5 μg), trimethoprim–sulfamethoxazole (10 μg) and linezolid (30 μg). All antibiotic disks were obtained from MAST ^®^ Company, U K. Methicillin susceptibility was determined using the cefoxitin E-test (Liofilchem, Italy) and oxacillin E-test (AB BIODISK, Sweden). *S. aureus* ATCC25923 was used as negative control and *S. aureus* ATCC43300 was used as positive control.

#### Genomic DNA extraction

Genomic DNA was extracted by Cinnaclon DNA extraction kit (Cinnaclon, Iran) based on manufacturer’s instruction. DNA was yielded and investigated by spectrophotometry using the Nanodrop (ThermoFishers, USA).

#### PCR for superantigen genes and screening for strains

The superantigen genes were amplified with specific primers listed by Schlievert and et al. [12] and Jarraud et al. [13] studies.

#### *agr* typing

Classification of *agr* system groups was based on the hyper variable domain of *agr* locus according to Soares et al. [14]. Duplex PCR was performed to type groups based on their product size.

#### Statistical analysis

Data were organized and analyzed using the Statistical Package for Social Sciences (SPSS) software, version 16. The correlation between phenotypic antibiotic pattern and *agr* locus, phenotypic antibiotic pattern and superantigens genes, superantigens genes and *agr* locus, sources of samples and *agr* locus in *S. aureus* isolates was evaluated by the Chi-square test and T-test. Statistical significance was set as a p-value of ≤ 0.05.

### Results

#### Prevalence of clinical isolates

**Fig. 1:**
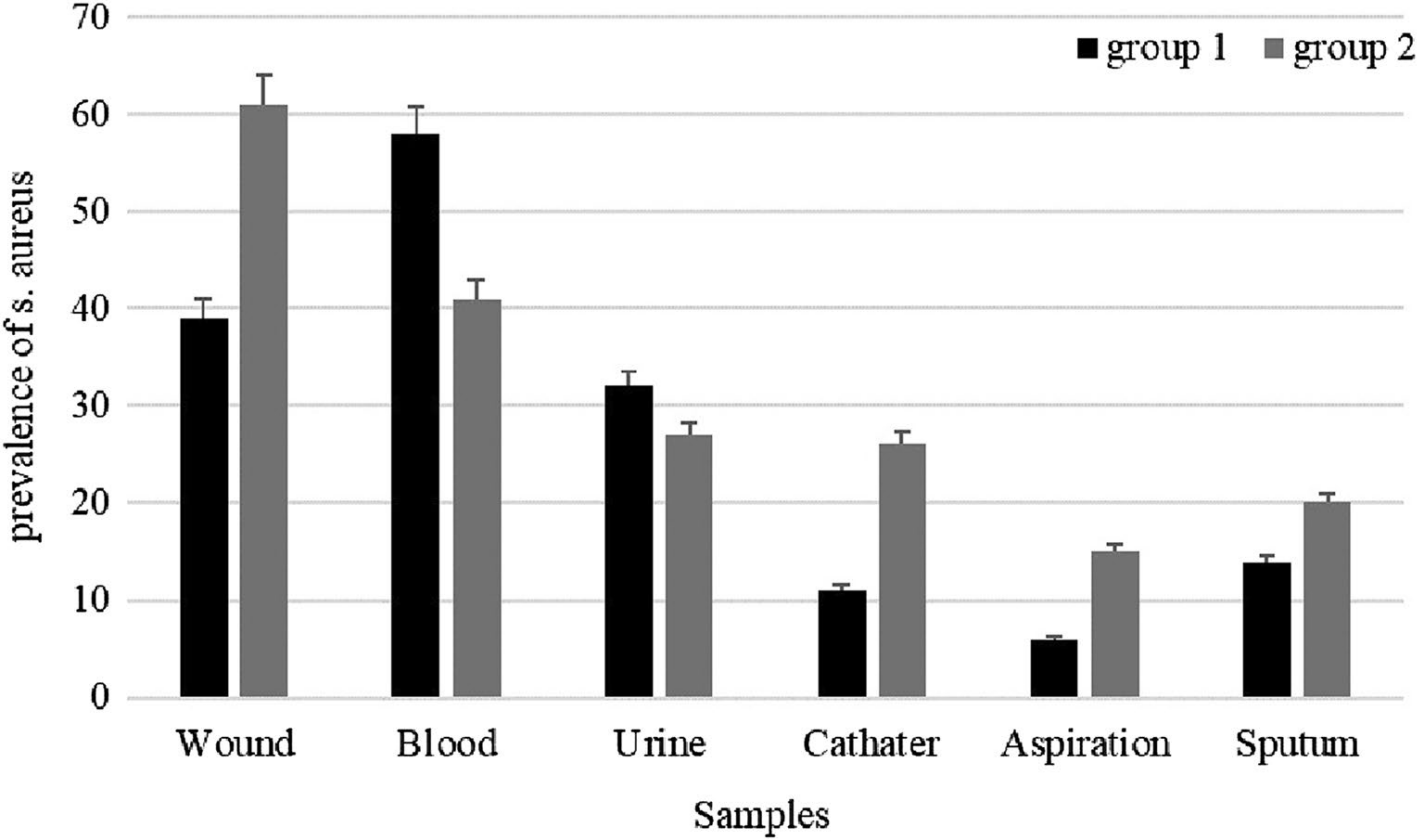
Distribution of different samples between Group 1 and Group 2 *Staphylococcus aureus*

Totally, 1009 clinical samples were collected from patients in *Hamedan* (Group 1) and *Sistan and baluchistan* (Group 2). 160 isolates were collected from Group 1 and 190 isolates were collected from Group 2. In Group 1, the most prevalent isolates were collected from blood 58 (36.25%). Also, in Group 2; most of the samples were isolated from wound 61 (31.10%) (Fig. 1).

#### Antibiotic resistance profiles and MIC

In Group 1, the most prevalent resistance was detected to penicillin (129, 80.62%) and to chloramphenicol (97 isolates, 60.62%). In addition, according to the results of of E-test strips, 2 (1.2%) isolates intermediate-resistance to vancomycin ≥ 3 µ/ml, 59 (36.87%) isolates resistant to oxacillin ≥ 4 µ/ml and 61 isolates (38.12%) resistant to cefoxitin ≥ 8 µ/ml were identified. Also, 61 (38.12%) MRSA strains were isolated from the clinical and screening samples.

In Group 2, penicillin and ciprofloxacin indicated as the highest resistance, 88.94% (169) and 63.68% (121) isolates, respectively. Moreover, based on the results of E-test strips, 4 (2.1%) isolates showed intermediate-resistance to vancomycin ≥ 3 µ/ml, 98 (51.50%) isolates resistant to oxacillin ≥ 4 µ/ml and 99 isolates (52.10%) isolates resistant to cefoxitin ≥ 8 µ/ml were identified. Also, 99 (52.10%) MRSA strains were isolated from the clinical and screening samples.

In Group 1, most of the MRSA samples were isolated from blood 66.66% (26 isolates) and wound 55.17% [32]. Whereas 0% (0 isolates) and 16.66% [1] of MSSA strains were detected in aspiration and sputum respectively. In Group 2, the most prevalent MRSA isolates were detected in blood 72.13% (44) and wound 46.34% [19], whereas MSSA isolates were identified in aspiration 26.66% [4] and sputum 10% [9] respectively, Table 1.

**Table 1:** Antimicrobial resistance profiles of MRSA, MSSA, and *S. aureus* isolates

Antibiotics	Group1						Group2						*S. aureus*					
	MRSA^a^ (*n* = 61)			MSSA^b^ (*n* = 99)			MRSA (*n* = 99)			MSSA (*n* = 91)			Group 1 (*n* = 160)			Group 2 (*n* = 190)		
	R^c^	I^d^	S^e^	R	I	S	R	I	S	R	I	S	R	I	S	R	I	S
Penicillin	61	0	0	68	0	31	99	0	0	70	0	21	129	0	31	169	0	21
Tetracycline	58	2	1	21	9	69	64	2	33	40	9	42	79	11	70	104	11	75
Clindamycin	49	4	8	35	9	55	69	4	26	28	2	61	84	13	63	97	6	87
Gentamicin	59	0	2	32	8	59	79	11	9	9	8	74	91	8	61	88	19	83
Ciprofloxacin	55	5	1	33	12	54	94	2	3	27	1	63	88	17	55	121	3	66
Erythromycin	47	3	11	22	9	71	58	6	35	50	0	41	50	9	82	108	6	76
Chloramphenicol	57	0	4	40	11	48	83	7	9	15	4	72	97	11	52	98	11	81
Linezolid	4	0	57	0	0	99	6	0	93	1	0	90	4	0	156	7	0	183
Trimethoprim/sulfamethoxazole	27	3	31	22	1	76	37	6	56	14	3	74	49	4	107	51	9	130
Rifampicin	7	0	54	4	0	95	8	0	91	1	0	90	11	0	149	9	0	181
MIC (µg/ml)																		
Vancomycin	0	2	59	0	0	99	0	4	95	0	0	91	0	2	158	0	4	186
Oxacillin	59	2	0	0	0	99	98	1	0	0	0	91	59	9	92	98	4	88
Cefoxitin	61	0	0	0	0	99	99	0	0	0	0	91	61	11	88	99	5	86

#### Superantigens genes profiles

Out of 190 *S. aureus* isolates of *Sistan and baluchistan,* 96 (50.5%) of *Zahedan,* 30 (15.7%) of *Khash* and 64 (33.6%) isolates of *Iranshahr* was collected. Also, out of 190 *Sistan and baluchistan* isolates, *seq* had the highest frequency and *edinB* had the lowest frequency, which were positive in 29 (15.2%) and 3 (1.5%) isolates, respectively. Moreover, out of 160 *S. aureus* isolates of *Hamedan,* the *seq* gene found in 22 (13.75%) isolates was more abundant. None of the isolates of *S. aureus* isolated from *Hamadan* had *etD*, *etA*, *etB*, *lukF*-*PV* and *lukE*-*lukD* genes. The prevalence of SAgs in female patients was higher than male patients. In addition, MDR strains also had the highest frequency of SAgs genes, Table 2.

**Table 2:** Prevalence of SAgs genes in *S. aureus* isolates from patients of Group 1 and Group 2

SAgs and toxins	Group 1 (*n* = 160)		Group 2 (*n* = 190)		MRSA		MSSA		Total in Group 1	Total in Group 2
	Female	Male	Female	Male	Group 1	Group 2	Group 1	Group 2		
*SEA*	5	12	11	14	11	20	6	6	17	26
*SEB*	1	10	9	10	7	11	4	8	11	19
*SEC*	3	2	5	16	6	19	0	2	5	21
*SED*	11	2	1	16	9	13	4	7	13	17
*SEE*	4	11	6	13	6	16	9	3	15	19
*SEl*-*G*	0	2	8	8	2	11	0	5	2	16
*SEl*-*H*	1	5	3	13	5	9	1	7	6	16
*SEI*	4	3	7	13	6	15	1	5	7	20
*SEl*-*J*	5	6	2	9	7	16	4	2	11	14
*SEl*-*K*	1	6	1	6	6	11	1	2	7	13
*SEl*-*L*	4	5	2	7	4	19	5	2	9	21
*SEl*-*M*	2	9	5	6	10	17	1	9	11	26
*SEl*-*N*	5	7	5	7	9	11	3	8	12	19
*SEl*-*O*	9	13	7	15	16	23	6	6	22	29
*SEl*-*Q*	8	13	9	12	19	17	2	2	21	19
*TSST*-*1*	0	1	4	10	1	9	0	5	1	14
*etA*	0	0	3	8	0	9	0	3	0	11
*etB*	0	0	3	6	0	8	0	1	0	9
*lukS*-*PVL*	0	2	2	4	2	5	0	3	2	8
*lukF*-*PV*	0	0	3	8	0	5	0	6	0	11
*lukE*-*lukD*	0	0	2	4	0	6	0	0	0	6
*edinA*	1	0	0	0	1	0	0	0	0	0
*edinB*	0	0	0	0	0	0	0	0	0	0
*edinC*	1	0	0	0	1	0	0	0	0	0
*mecA*	49	12	67	32	61	99	0	0	61	99

#### *agr* typing

In Group 1 out of 160 isolates of *S. aureus*, 104 (65%) *agr* positive and 56 (35%) *agr* negative were detected. The frequency of *agr* locus was identified as 27 (25.96%) *agrA*, 49 (47.11%) isolates *agrB*, 17 (16.34%) *agrC* and 11 (10.57%) *agrD*. In Group 2, among 190 isolates of *S. aureus*, 132 (69.47%) were positive for *agr* and 58 (30.52%) negative for *agr*. Also, the frequency of *agr* locus was detected as follows: *agrA* in 39 (29.54%) *agrB* in 55 (41.66%), *agrC* in 29 (21.96%) and *agrD* in 9 (6.81.96%), Additional file 1: Tables S1, S2.

#### Statistical analysis

In this study, using t-test and Chi^2^, there was a significant relationship between the SAgs and *agr* locus frequency. And also, a significant relationship was found between phenotypic antibiotic resistance and *mecA*.

### Discussion

*Staphylococcus aureus* as a threatening agent in hospitals and societies has a diverse range of strategies including antibiotic resistance, virulence factors and precise regulatory systems which accurately control and synchronize pathogenicity [15]. Therefore, in order to find the relationship among *agr* types, superantigens production and resistance in MRSA strains, we investigated clinical isolates collected from two different regions of Iran, Hamadan (Group 1) and Sistan and Baluchistan (Group 2).

Among 160 isolates of Group 1 and 190 isolates of Group 2, agrII was the most prevalent type. Strains with agr typeII indicated the highest superantigens production in both groups (p < 0.05). SEl-Q, SEl-O and SEB showed the highest prevalence in Group 1 whereas in Group 2 SEl-O and SEA were observed as the most widespread ones. Also, TSST-1, exfoliative toxins and pantone valentine toxins were detected only in Group 2. As a common feature between both groups, superantigen production is more prevalent in MRSA strains than MSSA ones (p < 0.05).

Regarding to the different frequency of antibiotic resistant and pathogenic strains in Groups 1 and 2, it is suggested that different climate conditions may cause extensive changes in resistance and pathogenicity of the bacterium. As MacFadden et al. [16], Singer et al. [17], and Kurenbach et al. [18] studies prove this notion, differences in the patterns of climate can lead to widespread changes in antibiotic resistance patterns. Consistent with our results, Zhang et al. [19] demonstrated the effects of various environmental conditions on antibiotic resistance and virulence factors in bacteria. Agr typing as a convenient virulence typing method could contribute to a more precise understanding of the pathogenesis and epidemiology of staphylococcal infections [20]. Consistent with Collery, Nowrouzian and Chini, superantigen production in *S. aureus* is directly correlated to *agr* type of isolates. In so-called studies, the most prevalent superantigens were observed in *agr* types I and III, while in our study agrII was the predominant one [21–24]. To explain, clonal differences of strains collected from different regions should be regarded. Based on Guijarro and khelissa studies, environmental clues influences on evolution processes of the organisms and consequently variety in characteristics of strains occurs [25–27]. Moreover, in MRSA strains *mecA* gene leads to some changes in virulence factors of the organism [28]. The activity of this gene affects some structural proteins such as *agr*, and *agr*-regulated SAgs such as TSST-1 and SEs, ETs and PVL are influenced [13]. As it is demonstrated in this study, there was a significant association between MRSA prevalence and superantigen production and interestingly superantigens which are regulated by *agr* system were predominated in Group 2. To illustrate, based on many studies, there is a direct relationship between presence of the *mecA* gene and the bacterial phenotypic resistance [29–31]. Studies by Vitali et al. [32], Duran et al. [33] have shown that the presence of the *mecA* gene could affect *Staphylococcus aureus* strains in terms of antibiotic resistance patterns. The results of antimicrobial resistance studies conducted in Group 2 were shown a high prevalence of resistance to antibiotics. Although multi-drug resistance strains were detected in both groups, MDR strains predominated in Group 2. As well, vancomycin intermediate *S. aureus* (VISA) strains in Group 2 were observed more than Group 1. Several factors could be involved in this difference containing age, gender, climatic conditions, food type and regional culture. According to Lundgren et al. [34], Norris et al. [35] and Wushouer et al. [36] which concluded that, cultural factor is one of the most important causes of antibiotic resistance. Consistent with the above mentioned studies, patients in Group 2 were more interested in taking different drugs, and patients in Group 1 showed less willingness to take medication.

In conclusion: a significant relationship between the SAgs frequency and *agr* locus in both groups has been indicated. Also, a substantial relevance has been found among phenotypic antibiotic resistance and *mecA* gene (p < 0.05). The production of superantigens in *S. aureus* plays an important role in the classification of *agr* locus, and this locus can affect differently in methicillin-resistant strains.

## Limitations

The results of this study suggest that the activity of various promoters and operons (PII, PIII and *egc* operon) in *S. aureus* is directly related to *agr* locus. It seems that SAgs play a role as checkpoints of dissemination. In the current study, collaboration of antibiotic resistance with superantigen production has been proved (p < 0.05). However, the accurate mechanism of such a relationship should be unraveled.

## Additional file

**Additional file 1: Table S1.** Characteristics of the *agr* allelic profiles of Group 1 *S. aureus*. **Table S2.** Characteristics of the *agr* allelic profiles of Group 2 *S. aureus*.

## Abbreviations

*agr*: accessory gene regulator
MRSA: methicillin resistant *S. aureus*
SAgs: superantigens
PBP2A: penicillin binding protein
SCCmec: the staphylococcal chromosomal cassette
VISA: vancomycin intermediate-level resistant
VRSA: vancomycin resistant isolates
PVL: Panton-Valentine leukocidin
TSST-1: toxic shock syndrome toxin-1
MGEs: mobile generic elements
CLSI: Clinical Laboratory Standards Institute
