# Supplementary material for: Association between the accessory gene regulator (agr) locus and the presence of superantigen genes in clinical isolates of methicillin-resistant Staphylococcus aureus
Source: BMC Res Notes. 2019 Mar 12;12:130. doi: 10.1186/s13104-019-4166-7 (PMC6419358; doi:10.1186/s13104-019-4166-7)
Supplement: Supplementary file 1 — Additional file 1: Table S1. Characteristics of the agr allelic profiles of Group 1 S. aureus. Table S2. Characteristics of the agr allelic profiles of Group 2 S. aureus. [file 13104_2019_4166_MOESM1_ESM.docx]

Table S1:

| Superantigen and  Toxins | MRSA^a^ | | | | | MSSA^b^ | | | | | Total Genes | Total MRSA | Total MSSA | p.value |
| --- | --- | --- | --- | --- | --- | --- | --- | --- | --- | --- | --- | --- | --- | --- |
|  | *agr* typing | | | | | *agr* typing | | | | |  |  |  |  |
|  | I | II | III | IV | ND | I | II | III | IV | ND |  |  |  |  |
| *SEA* | 2 | 3 | 2 | 0 | 4 | 1 | 2 | 1 | 0 | 2 | 11 | 7 | 4 | p<0.05 |
| *SEB* | 1 | 4 | 1 | 0 | 1 | 0 | 2 | 1 | 0 | 1 | 9 | 6 | 3 | p<0.05 |
| *SEC* | 0 | 1 | 0 | 0 | 0 | 0 | 0 | 0 | 0 | 0 | 1 | 1 | 0 | p<0.05 |
| *SED* | 4 | 2 | 0 | 2 | 1 | 1 | 2 | 0 | 0 | 0 | 11 | 8 | 3 | p<0.05 |
| *SEE* | 1 | 4 | 0 | 0 | 1 | 1 | 5 | 1 | 0 | 2 | 12 | 5 | 7 | p<0.05 |
| *SEl-G* | 2 | 0 | 0 | 0 | 0 | 0 | 0 | 0 | 0 | 0 | 2 | 2 | 0 | p<0.05 |
| *SEl-H* | 1 | 2 | 0 | 0 | 2 | 0 | 1 | 0 | 0 | 0 | 4 | 3 | 1 | p<0.05 |
| *SEI* | 2 | 2 | 1 | 0 | 1 | 0 | 1 | 0 | 0 | 0 | 6 | 5 | 1 | p<0.05 |
| *SEl-J* | 2 | 3 | 1 | 0 | 1 | 1 | 3 | 0 | 0 | 0 | 10 | 6 | 4 | p<0.05 |
| *SEl-K* | 3 | 3 | 0 | 0 | 0 | 0 | 1 | 0 | 0 | 0 | 7 | 6 | 1 | p<0.05 |
| *SEl-L* | 2 | 2 | 0 | 0 | 0 | 0 | 2 | 1 | 3 | 0 | 9 | 4 | 5 | p<0.05 |
| *SEl-M* | 2 | 5 | 1 | 1 | 1 | 0 | 1 | 0 | 0 | 0 | 10 | 9 | 1 | p<0.05 |
| *SEl-N* | 1 | 5 | 0 | 0 | 3 | 0 | 1 | 2 | 0 | 0 | 9 | 6 | 3 | p<0.05 |
| *SEl-O* | 5 | 7 | 2 | 0 | 2 | 0 | 2 | 1 | 2 | 1 | 18 | 14 | 4 | p<0.05 |
| *SEl-Q* | 3 | 9 | 1 | 3 | 3 | 0 | 2 | 0 | 0 | 0 | 18 | 16 | 2 | p<0.05 |
| *TSST-1* | 1 | 0 | 0 | 0 | 0 | 0 | 0 | 0 | 0 | 0 | 1 | 1 | 0 | 0 |
| *etA* | 0 | 0 | 0 | 0 | 0 | 0 | 0 | 0 | 0 | 0 | 0 | 0 | 0 | 0 |
| *etB* | 0 | 0 | 0 | 0 | 0 | 0 | 0 | 0 | 0 | 0 | 0 | 0 | 0 | 0 |
| *lukS-PVL* | 1 | 1 | 0 | 0 | 0 | 0 | 0 | 0 | 0 | 0 | 2 | 2 | 0 | 0 |
| *lukF-PV* | 0 | 0 | 0 | 0 | 0 | 0 | 0 | 0 | 0 | 0 | 0 | 0 | 0 | 0 |
| *lukE-lukD* | 0 | 0 | 0 | 0 | 0 | 0 | 0 | 0 | 0 | 0 | 0 | 0 | 0 | 0 |
| *edinA* | 1 | 0 | 0 | 0 | 0 | 0 | 0 | 0 | 0 | 0 | 1 | 1 | 0 | 0 |
| *edinB* | 0 | 0 | 0 | 0 | 0 | 0 | 0 | 0 | 0 | 0 | 0 | 0 | 0 | 0 |
| *edinC* | 0 | 1 | 0 | 0 | 0 | 0 | 0 | 0 | 0 | 0 | 1 | 1 | 0 | 0 |

Table S2:

| Superantigen and  Toxins | MRSA | | | | | MSSA | | | | | Total Genes | Total MRSA | Total MSSA | p.value |
| --- | --- | --- | --- | --- | --- | --- | --- | --- | --- | --- | --- | --- | --- | --- |
|  | *agr* typing | | | | | *agr* typing | | | | |  |  |  |  |
|  | I | II | III | IV | ND | I | II | III | IV | ND |  |  |  |  |
| *SEA* | 4 | 14 | 3 | 2 | 3 | 1 | 2 | 1 | 0 | 3 | 27 | 23 | 4 | p<0.05 |
| *SEB* | 3 | 5 | 2 | 0 | 1 | 2 | 2 | 1 | 0 | 4 | 15 | 10 | 5 | p<0.05 |
| *SEC* | 5 | 7 | 2 | 1 | 4 | 0 | 2 | 0 | 0 | 0 | 17 | 15 | 2 | p<0.05 |
| *SED* | 5 | 4 | 2 | 1 | 1 | 2 | 2 | 0 | 0 | 3 | 16 | 12 | 4 | p<0.05 |
| *SEE* | 4 | 9 | 0 | 0 | 3 | 1 | 2 | 0 | 0 | 0 | 16 | 13 | 3 | p<0.05 |
| *SEl-G* | 7 | 4 | 0 | 0 | 0 | 1 | 3 | 0 | 1 | 0 | 16 | 11 | 5 | p<0.05 |
| *SEl-H* | 4 | 1 | 1 | 0 | 3 | 1 | 3 | 1 | 0 | 2 | 11 | 6 | 5 | 0 |
| *SEI* | 6 | 3 | 0 | 2 | 4 | 4 | 1 | 0 | 0 | 0 | 16 | 11 | 5 | p<0.05 |
| *SEl-J* | 7 | 2 | 1 | 0 | 5 | 1 | 1 | 0 | 0 | 0 | 12 | 10 | 2 | p<0.05 |
| *SEl-K* | 9 | 2 | 1 | 0 | 0 | 0 | 2 | 0 | 0 | 0 | 14 | 12 | 2 | p<0.05 |
| *SEl-L* | 7 | 4 | 1 | 0 | 7 | 0 | 2 | 0 | 0 | 0 | 14 | 12 | 2 | p<0.05 |
| *SEl-M* | 7 | 9 | 1 | 0 | 1 | 5 | 1 | 1 | 0 | 2 | 24 | 17 | 7 | p<0.05 |
| *SEl-N* | 6 | 3 | 0 | 1 | 1 | 3 | 1 | 1 | 0 | 3 | 15 | 10 | 5 | p<0.05 |
| *SEl-O* | 7 | 9 | 1 | 0 | 6 | 4 | 1 | 0 | 0 | 1 | 22 | 17 | 5 | p<0.05 |
| *SEl-Q* | 8 | 3 | 2 | 1 | 3 | 2 | 0 | 0 | 0 | 0 | 16 | 14 | 2 | p<0.05 |
| *TSST-1* | 6 | 2 | 1 | 0 | 0 | 1 | 3 | 0 | 0 | 1 | 13 | 9 | 4 | p<0.05 |
| *etA* | 5 | 2 | 1 | 0 | 1 | 2 | 1 | 0 | 0 | 0 | 11 | 8 | 3 | p<0.05 |
| *etB* | 4 | 3 | 0 | 1 | 0 | 1 | 0 | 0 | 0 | 0 | 9 | 8 | 1 | 0 |
| *lukS-PVL* | 1 | 3 | 0 | 0 | 1 | 1 | 2 | 0 | 0 | 0 | 7 | 4 | 3 | p<0.05 |
| *lukF-PV* | 1 | 2 | 0 | 0 | 2 | 1 | 2 | 0 | 0 | 3 | 6 | 3 | 3 | 0 |
| *lukE-lukD* | 1 | 4 | 0 | 0 | 1 | 0 | 0 | 0 | 0 | 0 | 5 | 5 | 0 | 0 |
| *edinA* | 0 | 0 | 0 | 0 | 0 | 0 | 0 | 0 | 0 | 0 | 0 | 0 | 0 | 0 |
| *edinB* | 0 | 0 | 0 | 0 | 0 | 0 | 0 | 0 | 0 | 0 | 0 | 0 | 0 | 0 |
| *edinC* | 0 | 0 | 0 | 0 | 0 | 0 | 0 | 0 | 0 | 0 | 0 | 0 | 0 | 0 |
